# Mechanochemical tumescentless endovenous ablation: final results of the initial clinical trial

**DOI:** 10.1258/phleb.2011.010100

**Published:** 2012-03

**Authors:** S Elias, J K Raines

**Affiliations:** * Columbia Vein Programmes, Columbia University, New York, NY; † University of Miami, Homestead, FL, USA

**Keywords:** chronic venous disease, endovenous techniques, ultrasound-guided sclerotherapy, varicose veins, venous insufficiency

## Abstract

**Objective:**

The purpose of this study was to assess the *safety* and *efficacy* of the ClariVein^®^ system that employs mechanochemical ablation of the great saphenous vein (GSV).

**Method:**

Patients eligible for ablation of the GSV underwent micropuncture access with only local anaesthesia to insert a 4 or 5 Fr sheath. The ClariVein^®^ catheter was placed through the sheath, the wire was extruded, and the distal tip of the wire positioned 2 cm from the saphenofemoral junction under ultrasound guidance. Catheter wire rotation was then activated for 2–3 seconds at approximately 3500 rpm. With the wire rotating, infusion of the sclerosant was started simultaneously with catheter pullback. The sclerosant used was 1.5% liquid sodium tetradecyl sulphate (Sotradecol^©^, Bioniche Pharma Group, Geneva, Switzerland).

**Results:**

Thirty GSVs in 29 patients were treated. All patients have reached six-month follow-up; the average number of postoperative days is 260. No adverse events have been reported. The Primary Closure Rate is 96.7%.

**Conclusion:**

Mechanochemical ablation appears to be safe and efficacious. The ClariVein^®^ technique eliminates the need for tumescent anaesthesia. The great majority of incompetent GSVs can be treated with this technique.

## Introduction

Over the last 10 years, numerous minimally invasive methods have been utilized to treat great and small saphenous vein incompetence.^1,2^ Most of these techniques involve percutaneous access, local anaesthesia, some form of ablation, and short operative times with relatively good safety and efficacy. The endothermal technologies require the use of tumescent anaesthesia prior to energy delivery and a generator to produce either laser or radiofrequency energy. Results have improved and complications have decreased as these techniques and technologies have evolved.^3^ In the modern era of endothermal ablation (after 2006), efficacy rates of long-term closure are reported at levels well above 97%.^4,5^ However, these methods currently still require tumescent anaesthesia which can be a source of patient procedural discomfort; further, this portion of the procedure is the steepest part of the physician learning curve.

Recent reports have evaluated ultrasound-guided foam sclerotherapy of the great saphenous vein (GSV).^6^ While foam sclerotherapy does obviate the necessity for tumescent anaesthesia, efficacy rates are lower than endothermal ablation and reported complication rates are higher.^7,8^ At present, it cannot be stated that foam sclerotherapy is as efficacious as endothermal ablation.

A new mechanochemical device, (ClariVein^®^, Madison, CT, USA), was developed to minimize the negative aspects of both endothermal ablation and ultrasound-guided sclerotherapy (UGS) for the treatment of saphenous incompetence, while incorporating the benefits of each. The advantages of this hybrid system are standard percutaneous access, endovenous treatment, local anaesthesia only (no tumescence anaesthesia) and a short procedure time. Since this system does not use thermal energy, the potential for nerve damage is minimized. The negative aspects eliminated by the hybrid procedure are: the need for tumescence anaesthesia required for endothermal ablation and lower efficacy rates for UGS. The mechanochemical method achieves venous occlusion utilizing a wire rotating within the lumen of the vein at 3500 rpm which abrades (i.e. injures) the intima to allow for better efficacy of the sclerosant. A liquid sclerosant (sodium tetradecyl sulphate) is concomitantly infused through an opening close to the distal end of the catheter near the rotating wire. These two modalities, mechanical and chemical, achieve venous occlusion results equal to endothermal methods.

**Figure 1:**
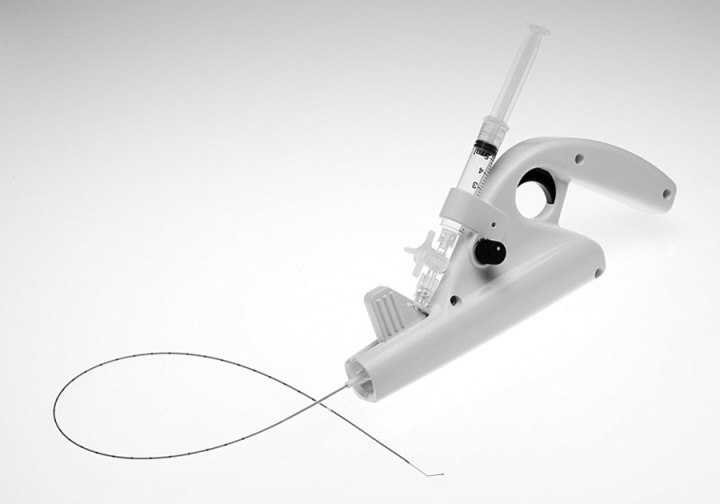
Picture of the ClariVein^®^ device

The entire device is for single use only and can be inserted through a 4 or 5 Fr sheath utilizing local insertion site anaesthesia only, without the need for tumescence anaesthesia. The system includes an infusion catheter, motor drive, stopcock and syringe (Figure 1). This report describes the initial human clinical trial in 30 limbs of mechanochemical ablation to treat GSV incompetence.

## Objectives

The purpose of this study was to assess the *safety* and *efficacy* of the ClariVein^®^ system which employs mechanochemical ablation of the GSV. Table 1 outlines the two primary objectives and the three secondary objectives of this study. Also given are the measures and measure definitions used to assess the objectives.

**Table 1:** Primary and Secondary objectives

Objective	Objective definition	Measures	Measure definition
Primary^1^	Determine overall safety of the ClariVein^®^ procedure	Adverse events and serious adverse events. This includes all clinical complications	Standard definitions of adverse events and serious advance events as defined by the FDA were used during the six-month postprocedure period
Primary^2^	Define recannulation of treated veins following the ClariVein^®^ procedure at six months	Primary Closure Rate at six months	Based on duplex ultrasound evaluation the closure of the treated vein is determined. Primary Closure Rate is calculated by dividing the number of closed veins at six months by the total veins treated (%). A continuous segment of 5 cm in length of treated vein is considered an open vein
Secondary^1^	Measures of pain associated with the ClariVein^®^ procedure and during follow-up	Pain levels reported by the patient	These data are gathered during the procedure and at all visits during the six-month postprocedure period
Secondary^2^	Listing of pain therapy during the ClariVein^®^ procedure and medication required during follow-up	Monitoring of medication required for pain	Monitoring of medication, dose, and timing for pain during the procedure and during the six-month postprocedure period
Secondary^3^	Identify eccymosis secondary to the ClariVein^®^ procedure	Degree of eccymosis as reported by the clinical staff using a simple scale	These data are gathered during the procedure and at all visits during the six-month postprocedure period. Possible answers ranged from No Eccymosis to Eccymosis over entire Length with Extension

FDA, Federal Drug Administration

## Materials and methods

ClariVein^®^ is an infusion catheter system designed to introduce physician-specified medications intravenously with simultaneous mechanical agitation into a patient's peripheral vasculature. Infusion is through an opening at the distal end of the catheter. Fluid delivery is enhanced by the use of a rotating dispersion wire to mix the infused fluid in the target vein and onto the vessel wall, as well as, abrade the venous intima. The dispersion wire extends through the catheter lumen. It is connected to an interface Cartridge Unit for connection to the 9 V DC battery motorized Handle Unit on the proximal end, which controls wire rotation. The Handle Unit also provides a grip and syringe holder to facilitate physician-controlled infusion. After purging with saline to ensure a closed system and prior to drug infusion, the wire plus catheter sheath is inserted into the vein percutaneously. The catheter sheath is retracted to expose the wire tip, which is positioned 2 cm from the saphenofemoral junction. The catheter motor is turned ON and, with the wire rotating and sclerosant infusing, the catheter is pulled down the vein at a rate of approximately 1–2 mm per second.

The wire that passes through the catheter is 304 V stainless steel; the configuration of the dispersion tip has been optimized for mechanochemical vein ablation. The wire is steerable and therefore will transverse most tortuous GSV segments.

### Protocol

#### Assessment

Patients underwent examination by a senior venous surgeon and ultrasound evaluation, and if deemed eligible for thermal ablation (laser or radiofrequency) of the GSV were considered for this Institutional Review Board (IRB) approved protocol. The assessment included evaluation of GSV reflux, Venous Clinical Severity Score (VCSS),^9^ CEAP (clinical, aetiological, anatomical and pathophysiological elements) classification^10^ and previous venous procedures.

Reflux was determined at the saphenofemoral junction in the standing position using the Valsalva manoeuvre or manual distal compression with rapid release. Reflux as documented by ultrasound extending for 0.5 seconds or longer was considered significant. CEAP Class 1 patients were excluded. Other exclusion criteria were the same as those for endothermal ablation and included: acute deep vein thrombosis (DVT), immobility, anticoagulation and GSV diameters > 12 mm.

#### ClariVein^®^ procedure

At the time of the procedure, patients were placed in the reversed Trendelenberg position on a procedure table. Repeat ultrasound examination was performed to confirm the important anatomic and haemodynamic parameters. This included imaging of the target vein for access, the saphenofemoral junction, perforators, tributaries, diameter and treatment length.

With the patient in the reversed Trendelenburg position, micropuncture access was obtained. Only local anaesthesia was used to insert the 4 or 5 Fr sheath. The ClariVein^®^ catheter was placed through the sheath, the wire was extruded and the distal tip of the wire positioned 2 cm from the saphenofemoral junction under ultrasound guidance. The patient was then rotated to a flat position for the remainder of the procedure. Catheter wire rotation was first activated for 2–3 seconds at the highest speed setting (approximately 3500 rpm). This creates venospasm which minimizes forward flow into the common femoral vein. With the wire continuing to rotate, infusion of the sclerosant was started simultaneously with catheter pullback. The sclerosant used was 1.5% liquid sodium tetradecyl sulphate (Sotradecol^©^). For this study all treated veins received 12 cc of 1.5% sclerosant. The sclerosant volume was independent of vein diameter and treatment length. The pullback rate was between 1.0 and 2.0 mm/second. No other concomitant procedures were performed (i.e. microphlebectomy or perforator ablation) so as to have the evaluation of safety and efficacy wholly dependent on mechanochemical ablation. All entered subjects underwent a completed procedure.

#### Postprocedure

When the catheter had traversed the entire treatment length and had been removed, the surgical team checked for GSV occlusion and patency of the common femoral vein using ultrasound. A 4′ and 6′ compression bandage was applied to the treated limb from the foot to the groin. This remained in place for 24 hours. The patients then applied a 15–20 mmHg thigh-high compression stocking continuously for the next 48 hours, except whilst showering. They then utilized the compression stocking only during the day for the next 10 days. Patient activity was not restricted. All forms of reasonable exercise were approved from the first postprocedure day. The patients were instructed to take only over-the-counter medication for discomfort.

#### Follow-up

**Figure 2:**
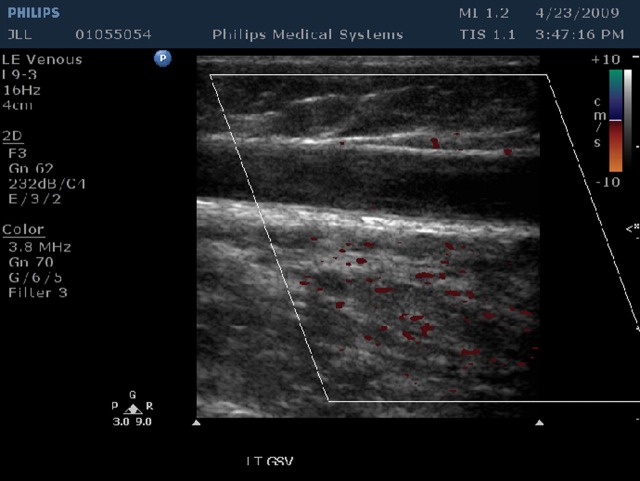
Ultrasound of closed GSV at one-week (no flow and no vein wall retraction). GSV, great saphenous vein

**Figure 3:**
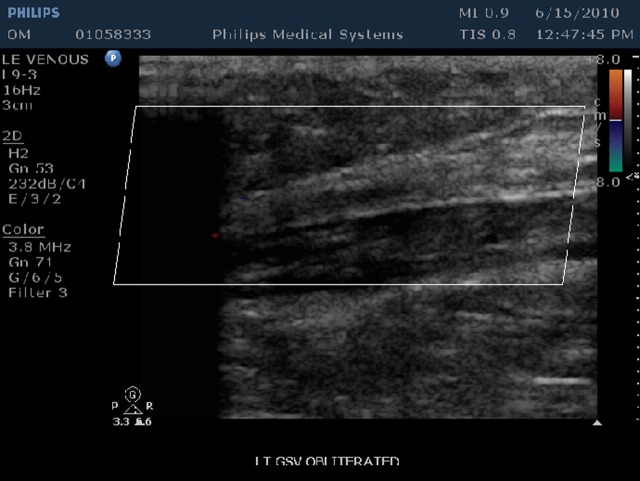
Ultrasound of closed GSV at 12 months (no flow and complete vein wall retraction). GSV, great saphenous vein

Follow-up visits at one week, one month, three months and six months were performed. At each visit an ultrasound study and clinical exam was performed. Occlusion and vein wall changes were documented (Figures 2 and 3).

## Results

Using the protocol described above, a single experienced venous surgeon treated 30 GSVs in 29 patients during the period 20 February 2009 and 17 July 2009 (±5 months). The average age of the patients was 54.3 years with a range of 31–90 years. Sixty percent of the subjects were women. The ablation procedures were limited to the GSV. In all cases comprehensive lower-extremity venous duplex examinations were performed to determine venous insufficiency and indications for the procedure. GSV reflux was carefully measured in the standing position. In all cases GSV reflux time was >2 seconds with a range of 2–5 seconds. In 47% of the operated limbs, veins other than the GSV, demonstrated a degree of reflux. This included the deep system, other superficial veins and perforating veins.

The average VCSS score was 4.5 with a range of 1–14. In all cases significant varicose veins were present. The six-point CEAP classification ranged from 2–4. In our series 77% were in Class 2 (varicose veins), 7% in Class 3 (varicose veins and oedema) and 16% in Class 4a (varicose veins with skin changes).

In our series the average diameter of the treated GSV was 8.1 mm with a range of 5.5–13 mm. The average treatment length was 37.5 cm with a range of 24–47 cm. The total procedure time averaged 14 minutes. The average total time for the ablation portion of the procedure was nine minutes with a range of 6–17 minutes. The catheter was in the active treatment mode, including pullback for an average of five minutes and 15 seconds. The range was three minutes and 40 seconds to six minutes and 45 seconds (pullback rate 1–2 mm/second). For this series no other vein segments were treated and no secondary treatment was administered. During the procedure patients did not complain of pain. Three minor thigh eccymosis were observed at levels where the rotating wire may have caught on a valve cusp or vein wall. No DVT nerve or skin injury occurred. No patient complained of parathesia, hypothesia or motor dysfunction on clinical examination.

To date, there are no patients lost to follow up. All patients have reached the six-month follow-up point. The average follow-up is 260 days with a range of 140–510 days. At this point only one vein has recanalized. This was the first case in this series. While there is ultrasound evidence of recanalization, the treated vein is not refluxing. The recanalization occurred between the one-week and one-month visits. This corresponds to a Primary Closure Rate of 96.7% at 260 days. Thus, any vein with a successful outcome at one-month has remained occluded at the six-month follow-up visit.

## Discussion

The first primary objective was to determine overall safety of the ClariVein^®^ procedure. Most investigators have found that the vast majority of adverse events associated with superficial vein closure occur at the procedure or within the first 30 days after the procedure. An exception is recanalization of the treated segment. Our first primary objective was to assess safety of the procedure. A total of 30 veins were treated in 29 patients. No adverse events or serious adverse events were recorded. Our definition of adverse event included new events not seen before the index surgical procedure, a pre-existing event that recurred with increased intensity or increased frequency subsequent to the index procedure and events which were present at the time of study entrance which became exacerbated. Our definition of serious adverse events included death, life-threatening events, any event which is disabling or incapacitating, events requiring prolonged hospitalization, any cancer and clinical chemistry results that are considered a major clinical concern. With no adverse events our findings suggest that the ClariVein^®^ procedure is safe based on up to six months of follow-up.

Our second primary objective was to determine efficacy. The measure we elected to use was Primary Closure Rate based on ultrasound. In our series, 29 of 30 veins are closed corresponding to a Primary Closure Rate of 96.7% at a mean follow-up of 260 days. Since this rate is comparable to the best published results for endovenous laser (EVL) and radiofrequency at the same follow-up, our assessment suggests the procedure is efficacious based on six months of follow-up.

The first and second secondary objective are associated with pain and required medication during the procedure and at follow-up. During the procedures (30 veins in 29 patients), no patient complained of pain and no medication in addition to the 1 cc injection at the cannulation site was requested. The third secondary objective is ecchymosis (bruising) secondary to the ClariVein^®^ procedure. Again since tumescent anaesthesia is not required, bruising was not noted in our patients. Three patients did have minor upper-thigh ecchymosis perhaps due to the rotating wire catching on the side branch or valve cusp. A gentle tension is required for release; this may cause a small tear in the vein wall.

Tumescent anaesthesia obviates the transfer of thermal energy to non-target tissues by creating a heat sink; further, it mechanically reduces the luminal diameter of the vein wall to better contact the indwelling items such as laser fibre or radiofrequency catheter. However, its placement requires multiple patient needle-sticks. Tumescent infusion is the steepest part of the learning curve for new practitioners. It is also the longest part of a short procedure; eliminating tumescent infusion is a desirable goal. Furthermore, thermal ablation (laser or radiofrequency) requires the acquisition of a generator and may be associated with a significant degree of postoperative pain and bruising.^3^ It should be acknowledged that the closure rates associated with thermal ablation are excellent, advances in radiofrequency catheters have reduced pain and bruising, and increased wavelengths in endovenous lasers have also reduced postoperative pain and bruising. The risk of thermal injury to nerve, muscle or skin is minimized.

However, the generator cost and the requirement for tumescent anaesthesia remain negative issues for this technology. ClariVein^®^ does not require a generator or the use of tumescent anaesthesia.

The risk of thermal injury to nerve, skin or muscle is eliminated. The GSV can be treated over its full length if clinically indicated without concern for nerve injury. This can also be extrapolated to treatment of the small saphenous vein.

The initial experience in patients described above using ClariVein^®^ clearly has been valuable on several fronts. While only 30 GSVs were treated, the age and age range of the patients and their presenting profiles mimic studies using other endovenous modalities. Actually, this study had a higher percentage of men than the average superficial venous ablation trial. The patients in this study were clear candidates for GSV treatment; however, they did not have advanced disease. The average VCSS was 4.5% and 77% of subjects were in CEAP Class 2. The average GSV supine diameter at the saphenofemoral junction was 8.1 mm (5.5–13 mm). This is the vein size published for many contemporary trials. Our average treatment length was 37.5 cm (24–47 cm) and is also consistent with current trial results.

## Conclusions

Mechanochemical ablation appears to be safe and efficacious. The technique eliminates the need for tumescent anaesthesia, a goal that both radiofrequency and laser technologies are currently exploring.^11^ The elimination of tumescent infusion decreases patient discomfort, shortens the physician learning curve and shortens procedure time by getting rid of the longest part of the relatively short procedure of endovenous ablation.

The great majority of incompetent GSVs can be treated with this technique. Veins which may not be candidates for mechanochemical ablation include those with previous thrombophlebitis which have recanalized and are incompetent. A few of those were attempted after the clinical trial. The rotating wire was found to tangle on the synechiae and trabeculae of the recanalized vein which limits mechanical treatment.

Follow-up in the initial 30 patients and subsequent patients will continue. In addition, a ClariVein^®^ registry has been initiated. Those patients treated by properly-trained physicians will be entered.

In conclusion, mechanochemical ablation utilizing the ClariVein^®^ device has good safety and appears to have good efficacy; the method should be considered as another viable alternative for the management of saphenous incompetence.

**Declarations:** JKR has worked as a consultant for Vascular Insights LLC. This research was sponsored in part by Vascular Insights LLC.
